# Excessive Innate Immunity Steers Pathogenic Adaptive Immunity in the Development of Theiler’s Virus-Induced Demyelinating Disease

**DOI:** 10.3390/ijms22105254

**Published:** 2021-05-17

**Authors:** Byung S. Kim

**Affiliations:** Department of Microbiology-Immunology, Northwestern University Feinberg School of Medicine, Chicago, IL 60611, USA; bskim@northwestern.edu; Tel.: +1-312-503-8693

**Keywords:** virus, demyelination, inflammation, Th cells, FoxP3^+^CD4^+^ T cells

## Abstract

Several virus-induced models were used to study the underlying mechanisms of multiple sclerosis (MS). The infection of susceptible mice with Theiler’s murine encephalomyelitis virus (TMEV) establishes persistent viral infections and induces chronic inflammatory demyelinating disease. In this review, the innate and adaptive immune responses to TMEV are discussed to better understand the pathogenic mechanisms of viral infections. Professional (dendritic cells (DCs), macrophages, and B cells) and non-professional (microglia, astrocytes, and oligodendrocytes) antigen-presenting cells (APCs) are the major cell populations permissive to viral infection and involved in cytokine production. The levels of viral loads and cytokine production in the APCs correspond to the degrees of susceptibility of the mice to the TMEV-induced demyelinating diseases. TMEV infection leads to the activation of cytokine production via TLRs and MDA-5 coupled with NF-κB activation, which is required for TMEV replication. These activation signals further amplify the cytokine production and viral loads, promote the differentiation of pathogenic Th17 responses, and prevent cellular apoptosis, enabling viral persistence. Among the many chemokines and cytokines induced after viral infection, IFN α/β plays an essential role in the downstream expression of costimulatory molecules in APCs. The excessive levels of cytokine production after viral infection facilitate the pathogenesis of TMEV-induced demyelinating disease. In particular, IL-6 and IL-1β play critical roles in the development of pathogenic Th17 responses to viral antigens and autoantigens. These cytokines, together with TLR2, may preferentially generate deficient FoxP3^+^CD25^-^ regulatory cells converting to Th17. These cytokines also inhibit the apoptosis of TMEV-infected cells and cytolytic function of CD8^+^ T lymphocytes (CTLs) and prolong the survival of B cells reactive to viral and self-antigens, which preferentially stimulate Th17 responses.

## 1. Theiler’s Virus-Induced Demyelinating Disease as an Infectious Model of Multiple Sclerosis

Multiple sclerosis (MS) is an immune-mediated neurological disease characterized by demyelination in the white matter of the brain and spinal cord [1]. Although the cause of MS is unknown, infectious agents may be involved in the initial infliction of tissue damage, leading to autoimmunity. A possible viral association is suggested by epidemiological studies [2,3] and by the detection of viral antigens and virus-specific antibodies in the majority of MS patients [4,5,6,7]. Several autoimmune and virus-induced models have been used to study the underlying mechanisms of this disease [8,9,10,11,12]. Among virus-induced models, Theiler’s murine encephalomyelitis virus (TMEV)-induced demyelination provides an excellent infectious model because of the similarities in the pathogenesis [9,10,13,14]. In addition, Theiler’s virus appears to be an emerging human virus group, called Saffold virus, which infects greater than 90% human populations [15,16,17]. Therefore, it is possible that MS is triggered by a combination of genetic prevalence and nonspecific bystander chronic viral infections resulting in the development of skewed pathogenic T cell types reactive to viral and self-antigens.

TMEV is a common enteric pathogen in mice and belongs to the family of picornavirus [18,19]. Two major subgroups of TMEV have been identified based on varying biological characteristics such as neurovirulence and antigenicity. The first subgroup of TMEV includes GDVII and FA viruses, which cause rapid and fatal encephalitis. The second subgroup, known as Theiler’s original viruses, includes the BeAn8386 and DA strains. Intracerebral inoculation of Theiler’s original viruses into susceptible mice causes a biphasic neurological disease [13,19,20,21]. Mice exposed to TMEV orally do not develop clinical symptoms and show reduced demyelinating disease after intracerebral inoculation of the virus, suggesting the infection route is important for the pathogenesis [22]. In fact, TMEV infection is spread widely via the fecal–oral route among wild mouse populations, yet these infected mice rarely develop clinical disease [23]. The early, acute phase displays flaccid limb paralysis and degeneration of neurons (poliomyelitis). The late phase exhibits chronic, inflammatory demyelination [20,21]. The BeAn strain, in contrast to the DA strain, is known to induce a clinically undetectable level of the early phase disease although it manifests as a severe late phase disease [13,20]. TMEV infection is also known to induce epilepsy and myocarditis depending on the injection sites and/or mouse strains [24,25]. However, this review focuses on the induction of demyelinating disease. Several hypotheses have been proposed to explain virus-induced demyelination. These include: (1) “bystander” damage of myelin [26,27] as a consequence of the host immune response against TMEV antigens; (2) induction of autoimmunity (via epitope spreading) to myelin proteins released by viral damage to the CNS [14,28,29]; and/or (3) induction and propagation of pathogenic antiviral and anti-self-immune responses by chronic overstimulations via pathogen pattern recognition receptors [14,30].

SJL/J (SJL) mice (H-2^s^) represent a prototypical susceptible mouse strain and C57BL/6 (B6) mice (H-2^b^) represent a prototypical resistant mouse strain against both viral persistence and the development of demyelinating disease [31,32]. Genetic studies of susceptibility to TMEV-induced demyelinating disease (TMEV-IDD) indicated that one of the important susceptibility loci is linked in the *H-2D* gene complex, suggesting the association with *H-2D*-restricted CD8^+^ T cell response [33,34]. However, CD8^+^ T cells generated in response to TMEV BeAn strain in susceptible SJL mice are restricted with the H-2K locus [35]. F1 (H-2^b/s^) of B6 and SJL mice are relatively resistant to TMEV-IDD and preferentially develop H-2D^b^-restricted CD8^+^ T cells of resistant B6 mice, not the H-2K^s^-restricted CD8^+^ T cells of susceptible SJL mice [27,36,37]. However, B6.S mice, similar to B10.S mice bearing H-2^s^, are relatively resistant to TMEV-IDD, indicating that other background genes may play a critical role in determining the susceptibility [38]. The major mouse strains used, susceptibility to TMEV, and their MHC and the background genes are shown in Table 1. The association with background genes is consistent with the previous genetic study indicating that TMEV persistence level in the central nervous system (CNS) is associated with non-MHC-linked genes on chromosomes 10, 14 and 18 [39,40,41]. In addition, TMEV persistence in the CNS appears to play an important role in the pathogenesis of demyelination [42,43,44,45,46,47,48]. However, the level of antiviral immunity is critical for the pathogenesis of demyelinating disease rather than the viral persistence levels as shown with TMEV-capsid transgenic mice, which are immunologically tolerant to the capsid antigens [49]. Therefore, it appears that viral persistence facilitates the production of continuous inflammatory cytokines and the consequent lasting pathogenic T cell responses for the development of TMEV-IDD. In this review, levels and types of innate and adaptive immune responses to TMEV will be analyzed in conjunction with the viral load to better understand the pathogenic mechanisms of virus-induced demyelinating disease.

## 2. Factors Affecting Permissiveness to TMEV Infection

### 2.1. Antigen-Presenting Cells

Many different cell types are permissive to TMEV infection, including neurons, oligodendrocytes, microglia, and astrocytes in the CNS, and dendritic cells, macrophages, and B cells of peripheral and infiltrating populations [30,50,51,52,53]. Non-professional antigen-presenting cells (APCs) in the CNS of TMEV-infected SJL mice, such as microglia and astrocytes, are capable of presenting antigens to both TMEV- and CNS autoantigen-specific T cell hybridomas and clones [54,55,56]. Furthermore, microglia and/or infiltrating macrophages in the CNS are a major cell population supporting viral persistence during chronic infection [56,57,58]. Virus replication is significantly higher in microglia from naïve SJL mice and the viral load is also greater in microglia from TMEV-infected SJL mice, compared to those cells from B6 mice [59]. In addition, differentiated/activated macrophages or astrocytes are much more permissive to TMEV infection/replication, providing the source of viral persistence in the CNS [60,61,62]. Consequently, the cytokine production level in microglia from SJL mice is higher compared to those from B6 mice. However, the levels of costimulatory molecule expression, and the ability to stimulate allogeneic T cells, are significantly lower in TMEV-infected SJL mice than in B6 mice [38,63]. These differences in the intrinsic properties of antigen-presenting cells for viral infection, replication and resulting innate cytokine production are likely to contribute to viral persistence, cellular infiltration to the CNS and consequent development of demyelinating disease. Moreover, these APCs, including macrophages/microglia, dendritic cells, and B cells in the CNS and periphery, appear to play additional important roles in stimulating T cells associated in the pathogenesis [30,63,64,65].

**Figure 1:**
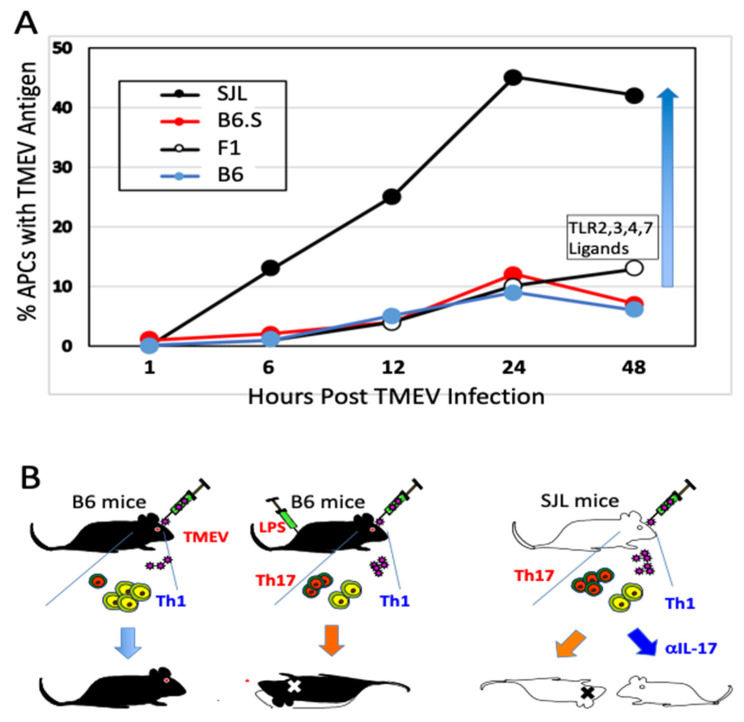
Permissiveness of antigen-presenting cells to Theiler’s murine encephalomyelitis virus (TMEV) infection correlates with susceptibility to the pathogenesis of demyelinating disease. (**A**) Susceptibility of antigen-presenting cells to TMEV infection/replication is directly associated with the development of chronic demyelinating disease. The elevated viral infection/replication leads to elevated inflammatory cytokine production (such as IL-6 and IL-1) which favors stimulation of pathogenic Th17 response over protective Th1 response. (**B**) Levels of Th17 determine the development of TMEV-induced demyelinating disease. Genetically resistant B6 mice induce strong Th1 and weak Th17 responses to TMEV infection. However, B6 mice receiving LPS develop strong Th17 and reduced Th1 responses, and become susceptible to TMEV-induced demyelination [48]. Susceptible SJL mice develop strong Th17 and weak Th1 responses. Injection of SJL mice with anti-IL-17A antibodies prevents the development of TMEV-induced demyelinating disease [65].

Further studies using B6.S mice, which carry the H-2^s^ with the resistant C57BL/6 background genes, are free from TMEV-IDD and display lower viral loads in the spinal cord compared to susceptible SJL mice [38]. Interestingly, viral infectivity and/or replication in glia and antigen-presenting cells (APCs) from TMEV-IDD resistant B6.S, B6, and F1(B6XSJL) mice are significantly lower compared to these SJL cells [37,59]. In vitro studies using APCs from B6.S and SJL mice show that TLR2, 3, 4, and 7-mediated signaling augment viral infection leading to the preferential differentiation of the pathogenic Th17 cell type in susceptible SJL mice [38,66]. Microglia and macrophages from susceptible SJL mice produce higher levels of IL-6 and IL-1 after TMEV infection compared to those cells from either B6, B6.S, or B10.S [67,68,69]. These observations indicate that the level of viral infectivity/replication controlled by non-MHC genes plays a critical role in the pathogenesis of chronic viral diseases. Taken together, these results strongly suggest that the viral replication levels in APCs critically affect the induction of protective vs. pathogenic Th cell types via the signaling of pattern recognition receptors for innate immune responses (Figure 1).

### 2.2. Role of Innate Immunity Associated with TMEV Infection

#### 2.2.1. Critical Roles of Pattern Recognition Receptors (TLRs and MDA-5)

**Figure 2:**
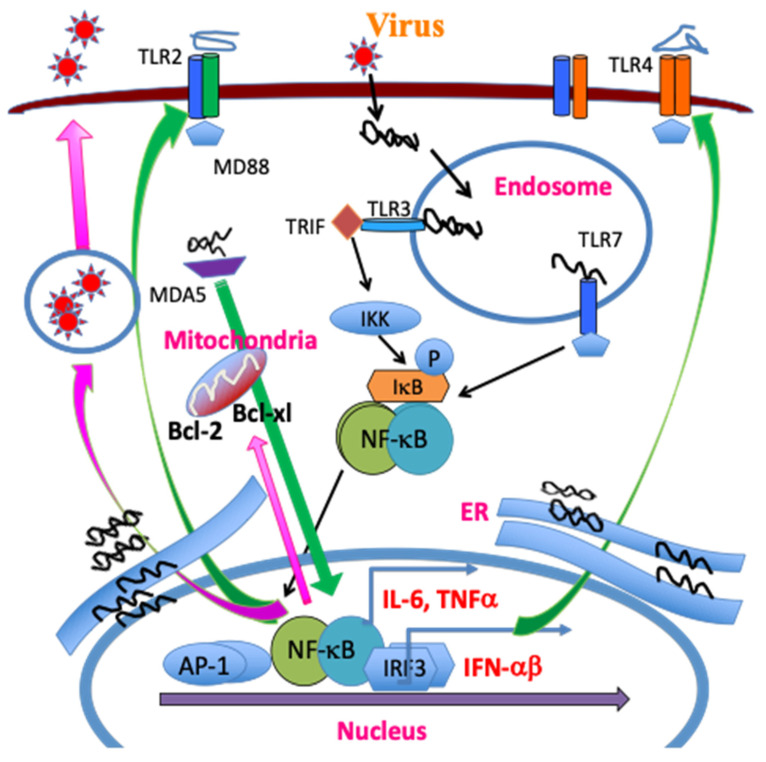
TMEV infection leads to release of a single stranded RNA genome and double stranded RNA replication intermediates in the endosome. The single RNA genome is recognized by TLR7, and the dsRNA intermediate, by TLR3. The TLR signaling activates NF-κB, AP-1 and IRFs, which in turn result in the production of various cytokines. MDA5 also recognizes viral messages leading to NF-κB activation. These activations lead to the additional expression and activation of TLR2/4, further activating NF-κB. The amplified NF-κB signaling promotes TMEV replication and the expression of Bcl-2 and Bcl-xL, which prevent virus-induced cellular apoptosis, increasing viral replication and cytokine production.

Mouse microglia express mRNA for TLR1–9 [58]. In addition, TLR stimulation activates the expression of MHC class II and costimulatory molecules, enabling the microglia to efficiently activate CD4^+^ T cells [58]. Infection of many different glial cells (neurons, microglia, oligodendrocytes, and astrocytes) and professional antigen-presenting cells (macrophages, dendritic cells, and B cells) with TMEV activates the production of various cytokines via TLR2, 3, 4, and 7 [30,38,70,71,72]. TMEV contains a single RNA genome recognized by TLR7 and the dsRNA intermediate by TLR3, and consequently TMEV infection activates NF-κB, AP-1, and IRFs, resulting in the production of various cytokines. These signals lead to further activation of NF-κB and increased production of various inflammatory cytokines such as IL-1β, IL-6, and IFNα/β, which augment the development of the pathogenic Th17 cell type [30,38,63,65,73]. In addition, the melanoma differentiation-associated gene 5 (MDA5) also recognizes viral messages leading to the activation of NF-κB and preferentially promotes the production of IFNα/β [74]. Interestingly, NF-κB signaling is necessary for viral replication and the expression of Bcl-2 and Bcl-xL, which prevent TMEV-induced cellular apoptosis extending viral replication and cytokine production [61,75]. Activation of chemokine and cytokine genes by TMEV is largely independent of the IFNαβ pathway and partly dependent on the dsRNA-dependent protein kinase (PKR) and MAP kinase pathways [73,76]. Interestingly, the activation of NF-κB is necessary for TMEV infection/replication [61] and, thus, the pattern recognition receptor-mediated cellular activation also serves as a mediator of viral persistence (Figure 2).

#### 2.2.2. NLRP3 Inflammasome

TLR-mediated signaling leads to the polymerization of the node-like receptor protein 3 (NLRP3) inflammasome, resulting in the activation of caspase-1 and the subsequent production of IL-1β and IL-18 [77,78,79]. The MDA-5 signaling also promotes the activation of NLRP3 [80,81]. Consequently, TMEV infection induces strong NLRP3 inflammasome and downstream prostaglandin E2 (PGE_2_) signaling in dendritic cells (DCs) and macrophages/microglia from susceptible SJL mice compared to the cells from resistant B6 mice [82]. Inhibition of PGE_2_ signaling using AH23848, an inhibitor of PGE_2_ receptor, decreases pathogenesis of TMEV-IDD and viral loads in the CNS, indicating the pathogenic role of PGE_2_ [82]. Furthermore, administration of IL-1β renders resistant B6 mice permissive to the development of TMEV-IDD, consistent with that the NLRP3 activation plays an important role in the pathogenesis [48,82]. The presence of a high IL-1β level elevates the pathogenesis by preferentially promoting the development of pathogenic Th17 responses [83]. These results suggest that the NLRP3 inflammasome signaling contributes to the pathogenesis of TMEV-induced demyelinating disease (Figure 2).

#### 2.2.3. Initial Chemokines and Cytokines

Viral and bacterial infections result in the production of chemokines and cytokines, which further activate additional cytokine and chemokine gene expression, promoting cellular infiltration and subsequent induction of adaptive immune responses [84,85,86]. Various chemokines and cytokines are produced in the CNS of mice after viral infections, including TMEV [87,88,89,90,91,92]. TMEV infection upregulates various chemokine gene expressions in glial cells and other antigen-presenting cells as early as 1–3 h after infection [51,93]. The infection of primary glial cells, including astrocytes and microglia, results in the activation of chemokine genes (CXCL1, CXCL2, CXCL10, CCL2, CCL3, CCL4, CCL5, CCL7, and CCL12) that are important in the initiation of an inflammatory response [93,94,95]. These results suggest that glial cells play critical roles in the development of, or protection from, TMEV-induced, immune-mediated inflammatory disease. Treatment with anti-CCL2 antibodies results in a significant decrease in the clinical disease progression, decreased CNS inflammation, and reduced viral load in the CNS [96]. This study suggests a protective role of CCL2 in the development of TMEV-IDD. In contrast to CCL2, both the lack of CXCL1 during TMEV infection and the excessive presence of this chemokine promote the pathogenesis of demyelinating disease. Therefore, a balance in the level of CXCL1 during TMEV infection is critically important in controlling the pathogenesis of demyelinating disease, although the level of CXCL1 produced is significantly higher in cells from susceptible SJL mice compared to that in cells from resistant BALB/c or B6 mice [95,97].

Proinflammatory cytokines, such as IL-1β, IL-6, IFNα/β, and TNFα, produced upon TMEV infection via TLR-mediated signals, further upregulate the production of chemokines [67,70,73,98]. In addition, TMEV infection upregulates the production of fibrin deposition, different adhesion molecules such as ICAM and VCAM, and endothelin-1 associated with blood–brain barrier permeability and cellular infiltration, which appear to participate in the pathogenesis of demyelinating disease [56,99,100,101,102]. TNF-α also appears to play an important pathogenic role in the development of TMEV-IDD because higher numbers of TNF-α producing cells are found in TMEV-infected susceptible mice [103]. However, TNF-α may also play a protective role because the absence of TNF-α worsens the pathology in TMEV-infected mice [104]. The signaling by NLRP3 inflammasome leads to the production of IL-1β and PEG_2_ [105]. TMEV infection activates NLRP3 via TLR signaling [82]. IL-1 and other inflammatory cytokines produced after TMEV infection are greater in susceptible mice compared to resistant mice [67]. A high IL-1 level plays a pathogenic role by elevating pathogenic Th17 responses, whereas a lack of IL-1 signals promotes viral persistence in the spinal cord due to insufficient T cell activation by elevating the production of inhibitory cytokines and regulatory molecules [48,83]. The inhibition of virus-induced PGE_2_ signaling results in decreased pathogenesis for TMEV-IDD, suggesting that the excessive activation of the NLRP3 inflammasome leading to the PGE_2_ signaling contributes to the pathogenesis [82]. The excessive PEG_2_ levels may prevent the T cell killing of target cells by inhibiting CD95L expression [106] and type I INF production [82]. TMEV infection upregulates the expression of PD-1 and PDL-1 in the CNS via type I IFN signaling in conjunction with the presence of IL-6 signaling [107,108].

TMEV infection results in the production of relatively high levels of Type I IFNs in various cell types from susceptible mice [73,109,110]. Type I IFNs stimulate T cell responses after TMEV infection by activating the expression of costimulatory molecules on APCs [30,63,111,112,113]. IFN-IR KO mice develop rapid fatal encephalitis accompanied with greater viral load and infiltration of immune cells of the CNS compared to the wild type mice [113]. The less efficient stimulation of virus-specific T cells in IFN-IR KO mice is attributable to the poor expression of costimulatory molecules on APCs. However, IFN-I also controls cellular infiltration to the CNS and shapes local T cell immune responses and B cell activation [30,63,113]. SJL DCs infected with TMEV induce rapid production of several different Type I IFNs (IFN-α1, IFN-α2, IFN-α4, IFN-α5, IFN-α6, IFN-α7, IFN-α9, IFN-α11, IFN-αβ, and IFN-β), and a type II IFN (IFN-γ) [63]. TMEV-infected DCs from susceptible mice produce higher levels of type I IFNs and IFN-γ compared to virus-infected DCs from resistant mice. The difference in the production of IFNs contributes to the significantly different virus-induced apoptosis, inhibition of development, and function of DCs [63]. The antiviral effect of Type I IFNs on TMEV replication is rather narrowly limited to just before viral infection. Therefore, the presence of high levels of Type IFNs in susceptible SJL mice may not necessarily be beneficial in controlling viral loads in the TMEV-induced demyelinating disease.

IL-6 and IL-1β play particularly important roles in the pathogenic T cell responses in the development of TMEV-induced demyelinating disease [65,83,113]. In addition, the IL-1β signal amplifies IL-6 production and activates CD4^+^ T cell expansion [48,114]. IL-6 participates in initiating and amplifying the pathogenesis of TMEV-induced demyelinating disease. In fact, mice lacking IL-6 signaling fail to develop demyelinating disease following TMEV infection [65,75]. However, the effect of IL-6 could be different depending on the TMEV strains because SJL mice receiving IL-6 prior to TMEV DA infection are free from the disease [115]. In addition, the presence of IL-6 is essential for the survival of mice infected with other viruses, such as influenza virus or lymphocyte choriomeningitis virus [116,117].

## 3. Role of Virus-Specific Adaptive Immunity in TMEV-Induced Demyelination

### 3.1. CD4^+^ T Cells

#### 3.1.1. Early Studies of CD4^+^ T Responses

**Figure 3:**
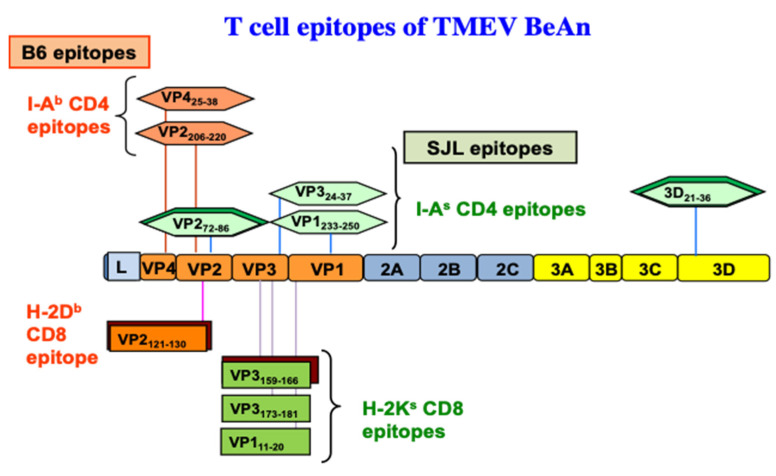
CD4^+^ and CD8^+^ T cell epitopes of the BeAn strain of TMEV restricted with MHC H-2^b^ and H-2^s^ haplotypes. The predominant epitopes are indicated by double boxes. One predominant H-2D^b^-restricted CD8 T cell epitope [135] and one predominant and two subdominant epitopes were found to be restricted with H-2K^s^ [35].

The demyelination induced after TMEV-inoculation is immune-mediated based on early study results indicating that the treatment of mice with anti-thymocyte, anti-Ia (MHC class II), or anti-CD4 antibodies delays the onset of disease [118,119,120], and that the level of the delayed-type hypersensitivity response specific for viral antigens correlates with the course of clinical signs of disease [27]. During chronic TMEV infection, susceptible mice display prolonged T cell immune responses toward viral antigens [13,121,122]. The association between susceptibility to TMEV-induced demyelinating disease (TMEV-IDD) and the MHC [34,121], in addition to the T cell receptor (TCR) β-chain genes [123,124], further supports involvement of T cell responses in the pathogenesis. Moreover, many studies have suggested that the CD4^+^ Th response is pathogenic in susceptible mice [125,126,127]. Alternatively, some studies indicate that CD4^+^ T cells confer protection from disease [128,129,130]. However, these early studies potentially include different CD4^+^ T cell subpopulations, including Th1, Th17, and Treg cells, which are functionally different. I-A^s^ restricted CD4^+^ T cell epitopes of TMEV in susceptible SJL mice have been identified: one major (VP2_72–86_) and two minor (VP1_233–244_, VP3_24–37_) epitopes on capsid proteins and one predominant epitope (3D_21–36_) on RNA polymerase [126,131,132,133]. Similarly, two predominant I-A^b^-restricted epitopes in resistant B6 mice have been found in the capsid region [134]. A diagram displaying the CD4^+^ T cell (I-A^s^ and I-A^b^) and CD8^+^ T cell epitopes (H-2K^s^ and H-2D^b^) of TMEV BeAn is shown in Figure 3.

#### 3.1.2. Utilization of Virus-Specific CD4^+^ T Cell Receptor Transgenic Mice

**Figure 4:**
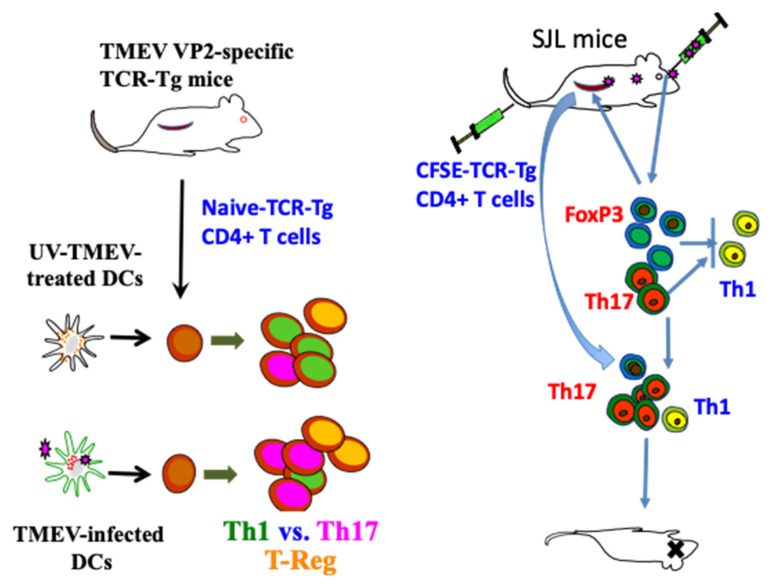
Utilization of TMEV VP2_72–86_-specific TCR-Tg mice in analyzing the development of CD4^+^ T cell subpopulations during TMEV infection [65,136]. These signals induce the production of IL-1, IL-6, IFNα/β, and TGF-β, which promote elevated induction of pathogenic FoxP3^+^ T-reg, Th17, and Tc17. Furthermore, IL-6 and IL-17, in addition to Tim and PDL-1, synergistically inhibit protective CD8^+^ T lymphocytes (CTL) function [107]. IFNα/β inhibits Th1 responses and elevates the expression of costimulatory molecules on antigen-presenting cells (APCs), which in turn promotes the induction of Th17 responses [63,113].

As CD4^+^ T cells include several subpopulations (Th1, Th17, and Treg), which have distinct functions in the development of antiviral immunity and inflammatory diseases, the availability of naïve undifferentiated virus-specific CD4^+^ T cells is critically important for investigating CD4^+^ T cell differentiation during TMEV infection. TCR transgenic mice specific for a major CD4^+^ T cell epitope (VP2_72–86_) of TMEV restricted with I-A^s^ (018030, SJL.Cg-Tg (TcraTcrbVP2) 1 Bkim/J from the Jackson Laboratory) have been used to investigate the differentiation of CD4^+^ T cell types during viral infection [65,136]. CD4^+^ T cells from VP2_72–86_-specific TCR-Tg mice in the absence of TMEV infection can be the source of naïve CD4^+^ T cells and the T cell differentiation could be investigated in the presence of virus-infected APCs (Figure 4). The differentiation of CD4^+^ T cell types were investigated by comparing T cell development in TMEV-infected TCR-Tg mice with those infected with UV-inactivated TMEV. The differentiation was also investigated following the adoptive transfer of CFSE-labeled naïve VP2 TCR-Tg CD4^+^ T cells into SJL mice followed by TMEV infection [136]. Alternatively, the differentiation of naïve TCR-Tg CD4^+^ T cells has been investigated in vitro in the presence of TMEV-infected DCs or UV-inactivated TMEV-infected DCs [65].

#### 3.1.3. Involvement of Th1 Cells

TMEV-specific Th1 cells producing IFN-γ mediate lysis of the virus-infected glial cells in a Fas-dependent mechanism [137]. Preimmunization of SJL mice with capsid-epitope peptides significantly increased capsid-specific CD4^+^ T cell numbers in the CNS during the early stages of viral infection [138]. These preimmunized mice delay the development of demyelinating disease in SJL mice, suggesting a protective role of capsid-reactive Th1 cells. Genetically resistant mice with deficiencies in the IFN-γ or its receptor genes fail to clear TMEV and develop extensive demyelinating disease [139,140]. Similarly, the intraperitoneal (i.p.) injection of susceptible mice with an IFN-γ-neutralizing monoclonal antibody (mAb) or of mice deficient in the Type I IFN receptor significantly accelerates the onset of disease [112,141,142]. The treatment of mice with anti-IFN-γ mAb does not reduce the delayed-type hypersensitivity (DTH) response to the virus and does not produce significant effects in the clinical course of disease, suggesting that DTH response may not reflect their Th1 response [141]. However, intracerebral administration of recombinant IFN-γ significantly accelerates the onset of TMEV-induced disease and the enhancing effect of IFN-γ is abrogated with treatment with anti-IFN-γ mAb [141]. Therefore, the level of IFN-γ appears to play a key role in the TMEV-induced inflammatory response and the perturbation of this cytokine may alter the course of demyelinating disease. IFN-γ is produced by natural killer (NK) cells, and CD4^+^ and CD8^+^ T cells, and thus, the effect of IFN-γ may not entirely reflect the function of the CD4^+^ T cell population. In addition, the presence of IFN-γ receptors on the CNS glia suggests the importance of the target cells in the function of IFN-γ during TMEV-induced demyelination [143]. The effects of preimmunization and tolerization with individual epitopes indicate that capsid-specific CD4^+^ T cells are protective, whereas viral RNA polymerase (3D_21–36_)-specific CD4^+^ T cells exacerbate the development of TMEV-induced demyelinating disease [133]. These results suggest the location and abundance of Th1 responses also play a role in the protection from the pathogenesis of TMEV-induced demyelinating disease.

#### 3.1.4. Role of Th17 Cells

Th17 cells producing IL-17, which are a distinct subset of CD4_+_ T cells, are involved in the pathogenesis of various autoimmune diseases [144,145,146,147]. Th17 cells preferentially develop in an IL-6-dependent manner after TMEV infection [65,75]. The presence of IL-6 is necessary for the development of Th17 responses and the pathogenesis of TMEV-IDD as shown with IL-6 KO mice [75]. Th17 cells promote the pathogenesis of chronic demyelinating disease by increasing viral persistence via upregulating antiapoptotic molecules and blocking target cell killing by cytotoxic T cells. Administration of the neutralizing antibody against IL-17 augments viral clearance and prevents the pathogenesis of TMEV-induced demyelinating disease [65]. The presence of IL-6 further synergistically enhances the antiapoptotic function of IL-17 function, which further facilitates the TMEV persistence in the CNS [75]. Conversely, IL-17 may also further induce IL-6, which amplifies the IL-17/IL-6 cytokine circuit [148]. The association of Th17 responses to the pathogenesis of TMEV-induced demyelinating disease has been confirmed using Th17-biased RORγt Tg mice with the B6 background, which render the development of TMEV-IDD with elevated Th17 responses, in contrast to the control B6 mice [149]. Using isolated DCs infected with live TMEV and UV-inactivated TMEV, it has been demonstrated that the development of Th17 cells is dependent on DCs infected with live TMEV producing various innate immune responses [65]. Thus, these results indicate a pathogenic role of Th17 cells in persistent TMEV infection and the pathogenesis of associated chronic inflammatory demyelinating diseases (Figure 4).

#### 3.1.5. Participation of FoxP3^+^ Regulatory T Cells

High levels of FoxP3+CD4+ T cells are present in the CNS of virus-infected mice as early as 3 d after viral infection [150,151]. The early induction of FoxP3^+^CD4^+^ T cells may depend on the function of the TCR2-mediated signal [152,153] upregulated after TMEV infection [72]. When FoxP3^+^CD4^+^ T cells were removed, viral loads in the CNS and the development of clinical signs were significantly reduced in susceptible SJL mice [150], but not in resistant C57BL/6 mice [154]. These results suggest that the presence of a high level of FoxP3^+^CD4^+^ T cells promotes the pathogenesis of demyelinating disease. However, as much as a two-fold higher proportion of FoxP3^+^CD4^+^ T cells in the CNS of virus-infected SJL mice displaced CD25^lo^ [136]. Thus, FoxP3^+^CD4^+^ T cells in the CNS may require further activation to be functional regulatory T cells [155,156,157]. High levels of CD25^-^FoxP3^+^CD4^+^ T cells were also observed in chronic hepatitis B virus-infected patients [158] and patients with systemic lupus [159]. CD25^lo^FoxP3^+^CD4^+^ T cells may lose FoxP3 expression and undergo trans-differentiation into pathogenic Th17 cells [160,161]. Therefore, it is conceivable that some or most of the CD25^lo^FoxP3^+^CD4^+^ T cells may be converted into pathogenic Th17 cells under the environment of abundant cytokines such as IL-6 and IL-1β in the CNS of TMEV-infected mice [65,83,136].

**Figure 5:**
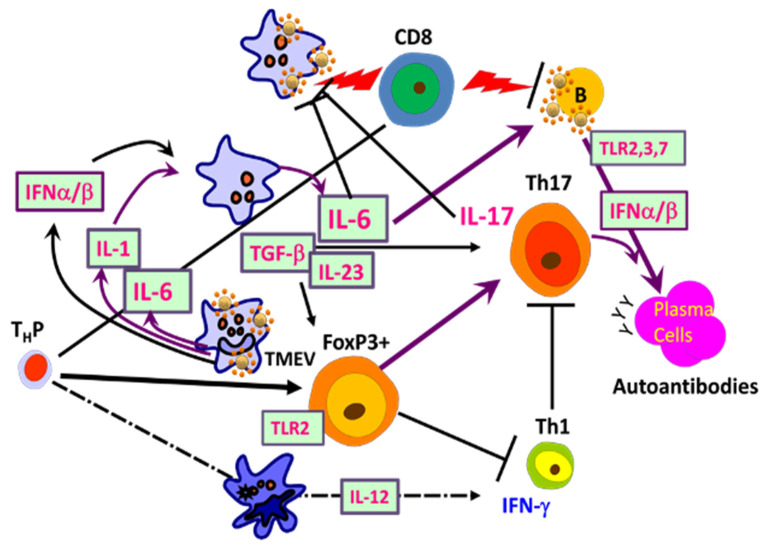
Interactions of adaptive immune responses to TMEV and autoantigens in susceptible mice. Infection of susceptible mice with neurotropic TMEV induces excessive levels of innate immune cytokines, including type I IFNs, IL-6, and IL-1, which promote inflammatory Th17 responses from FoxP3^+^ T-reg cells over protective Th1 responses, leading to high viral loads in the CNS. Various glias and antigen-presenting cells (APCs), including B cells, are permissive to the viral infection and participate in the innate immune responses. FoxP3^+^ T cells and Th17 cells are involved in the inhibition of protective CD8^+^ T cell function, which further elevates the viral persistence and pathogenesis of demyelination. B cells are also activated and stimulated to produce elevated levels of antibodies. Such high viral loads and innate cytokines, in addition to adaptive immune responses in the CNS, lead to CNS tissue damage releasing sequestered autoantigens.

In many chronic viral infections, including TMEV, FoxP3^+^CD4^+^ T cells appear to contribute to the pathogenesis by inhibiting protective T cell function and consequently promoting viral persistence [150,162]. In contrast, FoxP3^+^CD4^+^ T cells may be beneficial in controlling acute viral infections [163,164]. CD25^lo^FoxP3^+^CD4^+^ T cells do not appear to display regulatory function [157]. After activation of CD25^lo^FoxP3^+^CD4^+^ T cells in vitro under experimental conditions with various cytokines and peptides [156,165,166], up to 60% of CD4^+^ T cells bear FoxP3 and CD25^hi^ [136]. The TMEV VP2-specific FoxP3^+^CD4^+^ T cells activated in vitro inhibit the production of IFN-γ but not IL-17 by VP2-specific CD4^+^ T cells [136]. In contrast, activated epitope-specific FoxP3^+^CD4^+^ T cells in MHV-infected mice efficiently inhibit the proliferation of the same epitope-specific CD4^+^ T cells [151]. The previous studies indicating the inhibition of proliferation of Th cells utilized FoxP3^+^CD4^+^ T cells generated in vivo from TMEV-infected mice [150]. In addition, MHV-specific FoxP3^+^ CD4^+^ T cells were stimulated in vitro with anti-CD3 and anti-CD28 antibodies instead of using the epitope peptide [151]. Therefore, activating antigens for FoxP3^+^ CD4^+^ T cells and Th cells, in addition to the heterogeneity of target Th cells during the inhibition assays, may be important. TMEV-infected mice treated with ex vivo-generated FoxP3^+^ Tregs at an early stage of viral infection worsened the clinical signs, whereas the mice treated with the Tregs at a later stage decreased immune cell recruitment in the CNS [167]. Nevertheless, VP2 epitope-specific FoxP3^+^CD4^+^ T cells preferentially inhibited the production of IFN-γ, but not IL-17, by the same epitope-reactive Th cells [136]. As virus-reactive IFN-γ-producing Th1 cells are protective but IL-17-producing Th17 cells inhibit Th1 development and cytotoxic T cell function [65,168], FoxP3^+^CD4^+^ T cells together with Th17 cells may promote the pathogenesis of TMEV-induced demyelinating disease in an epitope-dependent manner (Figure 5).

### 3.2. Roles of CD8^+^ T Cells in the Pathogenesis of TMEV-IDD

#### 3.2.1. Role of Tc1

As demyelination is closely linked to viral persistence [42,43,44], TMEV-specific cytotoxic CD8^+^ T cells producing IFN-γ and perforin (Tc1) are likely to play an important role in protection and/or resistance [39,169,170]. TMEV-specific cytotoxic CD8^+^ T lymphocytes (CTL) appear to damage virus-infected, myelin-producing oligodendrocytes and other cell types [171,172,173,174]. Many investigations further confirmed the role of Tc1 cells by antibody-mediated CD8^+^ T cell depletion [175], and using Class I deficient mice [176,177,178]. Rodriguez and his colleagues proposed that CD8^+^ T cells are necessary for the manifestation of clinical symptoms using the DA strain of TMEV [169,173]. However, β_2_M-deficient or perforin-deficient mice on a resistant background are susceptible to both demyelination and clinical disease [170,176,177,178]. Furthermore, β_2_M-deficient mice with the susceptible SJL background displayed similar exacerbation of TMEV-IDD [179]. These results indicated a protective role of CD8^+^ T cells in the development of TMEV-induced demyelinating disease in both resistant and susceptible mice. Moreover, the presence of a high level of CTL in resistant mice and a low level in susceptible mice [180], and the resistance to TMEV-IDD in susceptible mice adoptively received CD8^+^ T cells [181], further support the protective function of CTL. In resistant B6 mice, the majority (50% to 70%) of CNS-infiltrating CD8^+^ T cells recognize VP2_121–130_ [182,183], and two minor populations (<10%) react with VP2_165–173_ and VP3_110–120_ capsid epitopes [184] based on the production of IFN-γ (Figure 3). Similarly, CNS-infiltrating CD8^+^ T cells of virus-infected SJL mice react with one predominant (VP3_159–166_,) and two subdominant capsid epitopes (VP3_173–181_, and VP1_11–20_) [35]. During the early stages of viral infection, a lower level of virus-specific CD8^+^ T cells in SJL mice was observed [184]. In addition, the resistance of (B6xSJL)F1 mice is associated with a higher level of the initial virus-specific H-2b-restricted CD8^+^ T cell responses compared to the H-2s-restricted CD8^+^ T cell responses [37]. These results further suggest that Tc1 cells play an important protective role in preventing TMEV-IDD by clearing viral loads from the CNS. There is, however, a possibility that certain CD8^+^ T cell populations play a pathogenic role, perhaps depending on epitope-reactivity or cytokine production, in TMEV-induced demyelination [171,172,173,185]. Similar CTL-mediated immunopathology was reported with the lymphochoriomeningitis virus (LCMV) and Coxsackie B virus in mice [186,187,188].

#### 3.2.2. Role of Tc17

Chronic inflammation promotes the induction of IL-17-producing CD8^+^ cells with reduced cytolytic function [189,190]. Therefore, it is conceivable that a subset of CD8^+^ T cells producing IL-17 may be associated with the pathogenesis of TMEV-IDD. To investigate the possible epitope-dependent function of CD8^+^ T cells in the protection and/or pathogenesis, a single amino acid substitution was introduced into the predominant viral epitope (VP3_159–166_) and/or a subdominant viral epitope (VP3_173–181_) of susceptible SJL/J mice by site-directed mutagenesis altering a single amino acid within the epitopes [191]. The resulting variant viruses (substituted at N160V, P179A, and N160V/P179A) failed to induce CD8^+^ T cell responses in the respective epitopes. TMEV (N160V and N160V/P179A) viruses, deficient in the predominant CD8^+^ T cell epitope, do not induce demyelinating disease [191]. The CD8^+^ T cells specific for the predominant VP3_159–166_ epitope identified by reactivity with the tetramers containing VP3_159–166_ showed strong cytolytic activity and produced high levels of IFN-γ. In contrast, VP3_173–181_-specific CD8^+^ T cells produced higher levels of transforming growth factor beta, interleukin-22 (IL-22), and IL-17 mRNA, and exhibited minimal cytotoxicity. Therefore, it is conceivable that differences in the functional avidity toward their cognate epitopes and/or the type of cytokines produced may affect the function of CD8^+^ T cell populations in an epitope-dependent manner. IFN-γ producing VP3_159–166_-specific CD8^+^ T cells with high cytolytic function, in contrast to low-cytolytic VP3_173–181_-specific CD8^+^ T cells, may be necessary to initiate the pathogenic process by destroying infected neurons and/or releasing sequestered autoantigens followed by Tc-17- and Th17-mediated inhibition of cytolytic function promoting viral persistence. Thus, both Th17 and Tc1 populations reactive to TMEV epitopes may cooperatively participate in the pathogenesis of virally induced demyelinating disease in an epitope-dependent manner [191].

### 3.3. Role of B Cells in the Development of TMEV-IDD

#### 3.3.1. Anti-TMEV Antibody Responses

The major neutralizing antibody epitopes of TMEV reside on VP1, and these epitopes may also be involved in virus-binding to cellular receptors [192,193]. The antibodies found in the spinal fluids of TMEV-infected mice [194] and in MS patients [195] displayed relatively limited heterogeneity. Furthermore, B cells producing antibodies to VP1 and VP2 proteins are detectable in the demyelinating lesions [196]. The plasma cells producing anti-TMEV antibodies were found primarily in perivascular infiltrates and in the meninges of the CNS parenchyma [197].

Antibodies from TMEV-infected mice also recognize several linear epitopes of 13–15 amino acid peptides or recombinant proteins representing the capsid proteins, and some of these antibodies display varying degrees of virus neutralization accompanying the protection from demyelinating disease development [122,198,199,200]. Anti-TMEV antibody responses during the early stage of viral infection play a protective role [49,201,202]. In addition, treatment of mice with the monoclonal anti-CD20 antibody accelerate the pathogenesis of TMEV-induced demyelinating disease, suggesting a protective role of the B cells [203]. However, antibodies to viral determinants appear to play a relatively minor role in the protection of mice from the pathogenesis of demyelinating disease compared to CD4^+^ Th1 and CD8^+^ T cells [122,201,202].

#### 3.3.2. B Cells as a Viral Reservoir and Their Role in T Cell Activation

An earlier study suggested that B cells are not permissive to TMEV infection [204]. However, a recent study showed as much as 40% of primary B cells in susceptible SJL mice are permissive to TMEV infection and replication [30]. TMEV-infected B cells expressed elevated levels of an activation marker, CD69. In addition, the expression of MHC class II and costimulatory molecules was upregulated in the infected B cells, accompanied by elevated levels of antigen-presenting function and antibody production [30]. The upregulation of these B cell activation markers induced with TMEV infection was replicated with the treatment of B cells with TLR ligands for TLR2, TLR3, TLR4, TLR7, and TLR9, suggesting that B cell activation after TMEV infection is associated with TLR signals [30]. These results are consistent with the previous findings that various TLR-mediated signals activate B cells to function as an important professional APC type involved in the activation of Th cell types [205,206,207]. The innate responses via TLRs following TMEV infection appear to trigger excessive production of IFN-α/β, IL-6, IL-1, and PGE_2_ in B cells of susceptible mice [30,208,209,210]. These innate immune responses following TMEV infection also activate B cells to produce antibodies and facilitate elevated T cell responses [30]. In addition, these cytokines may, in turn, further enhance the viral persistence by inhibiting apoptosis of TMEV-infected B cells and protective Th1 cell responses, while enhancing pathogenic Th17 responses [65,82] (Figure 5). Furthermore, TMEV-infected B cells form a germinal center-like structure with close proximity of T cells in the CNS, which may promote the induction of pathogenic T cells, such as Th17 and FoxP3^+^ Treg, recognizing not only viral determinants but also autoantigens [30].

## 4. Development of Autoimmune Responses during TMEV-IDD

Early studies indicated that antibody responses to self-antigens including myelin basic protein (MBP) in the CNS were induced during chronic infection with TMEV [28,30,211]. Similarly, Th cell responses to myelin-associated autoantigens in the CNS were also previously identified during chronic infection with TMEV [14]. In addition, TMEV-infected SJL mice displayed a CD8^+^ cytotoxic T cell population apparently recognizing both viral determinant and autoantigen capable of causing CNS degeneration [212]. Thus, chronic TMEV infection appears to induce various antibody, CD4^+^ T cell, and CD8^+^ T cell responses to CNS autoantigens. It has previously been proposed that the presence of molecular mimicry between viral determinants and autoantigens leads to the cross-reactive antibody and T cell responses following TMEV infection [213]. Alternatively, it is possible that sequestered autoantigens to the CNS may be released following TMEV-induced tissue damage and these released autoantigens induce such autoreactive antibody and CD4^+^ T cell responses [30,214]. However, rapidly accelerated production of the same autoantibodies was observed in systemic lupus erythematosus-prone mice, such as NZBWF1 and BXSB male mice, following infection with TMEV or Coxsackie virus [30]. These results strongly suggest that the autoimmune responses induced after TMEV infection reflect virus-induced TLR-mediated polyclonal activation of pre-existing autoimmune cells in a high frequency. In fact, ligands of various TLRs lead to the activation of B cells and T cells similar to TMEV infection [30]. The potential role of these autoimmune responses in the development of TMEV-induced demyelinating disease is not yet clear. As virus-reactive T cell responses are necessary to induce TMEV-induced demyelinating disease [49,55,65,191], the initial immune-mediated tissue damages of virus-infected cells may be a prerequisite for the pathogenesis of disease. Nevertheless, these autoimmune responses may participate in further damaging the related tissues and some may be involved in the protection, as previously suggested [215,216,217].

## 5. Shaping Adaptive Immune Responses by Innate Immunity after TMEV Infection

### 5.1. Excessive Innate Immunity Initiates the Pathogenesis of TMEV-IDD

The cytokine levels produced after TMEV infection are significantly greater in the glial cells and APCs of susceptible SJL mice than in those of resistant mice [38,59,67]. TLR-mediated signaling plays an important role in the induction of innate cytokine responses [30,70,71,72]. In particular, TMEV infection induces the high levels of IL-1β, IL-6, IFNα/β, and TNFα via TLR-mediated signals in cells from susceptible mice compared to those from resistant mice [67,70,73]. Treatment of susceptible mice with poly IC, a ligand of TLR3, prior to TMEV infection exacerbates disease development, whereas this treatment after viral infection decreases the disease development [66]. Excessive levels of cytokines such as IL-6 and IL-1β produced via TLR3 signaling prior to viral infection hinder the induction of protective IFN-γ-producing CD4^+^ and CD8^+^ T cell populations. Treatment of resistant B6 mice with LPS, a ligand of TLR4, or IL-1β, caused the mice to develop TMEV-induced demyelinating disease [48]. Similarly, susceptible SJL mice infected with a low pathogenic strain of TMEV developed a full-blown demyelinating disease after treatment with LPS [218]. Therefore, the excessive production of IL-6 and IL-1β appears to be mainly responsible for the development of demyelinating disease via preferential differentiation of pathogenic Th17 cells [65,83]. In addition, DCs treated with UV-inactivated TMEV predominantly induce protective Th1 responses in vitro, whereas DCs infected with live TMEV preferentially mount pathogenic Th17 responses [65]. The initial status of virus-specific CD4+ T cell differentiation appears to play an important role in the pathogenesis of disease, because adoptive transfer of VP2-primed VP2-TCR-Tg CD4+ T cells into naive SJL mice, in contrast to the naïve T cells, provides protection from TMEV-induced demyelinating disease [136]. In addition, IL-6 and IL-17, which are the cytokines produced by Th17, synergistically promote the survival of virus-infected cells by preventing cellular apoptosis and CTL-mediated cytolysis [75]. Furthermore, upregulated production of IL-6 results in the expression of higher levels of PD-1 and PDL-1, which inhibit CTL function of CD8^+^ T cells [107]. In addition, poly IC-pretreated mice displayed elevated PDL-1 and regulatory FoxP3^+^ CD4^+^ T cells in the CNS, whereas poly IC-post-treated mice expressed reduced levels of PDL-1 and FoxP3^+^ CD4^+^ T cells [66,107]. A high level of IL-1β, which amplifies IL-6 production [48,114], elevated pathogenic Th17 responses [83]. However, the lack of IL-1 signals promoted viral persistence due to insufficient T cell activation [83]. Therefore, the timing and balance of IL-1 signaling must be important for the protection from TMEV-induced demyelinating disease.

### 5.2. Viral Load and Persistence

Both immune response and viral persistence in the CNS appear to play an important role in the pathogenesis of demyelination [45,46,47,48]. The level of anti-TMEV immunity, rather than the viral persistence level, is important for the pathogenesis of demyelinating disease, as shown with P1-transgenic mice (Table 1) which are immunologically tolerant to all of the capsid proteins of TMEV [49]. The P1 region of TMEV encodes the major CD4^+^ and the CD8^+^ T cell epitopes (Figure 3). Despite the drastically increased viral loads in the CNS, SJL P1-Tg mice developed significantly reduced antiviral immune responses and less severe demyelinating disease, suggesting that TMEV persistence is required but not sufficient to induce the disease [49]. Therefore, the level of pathogenic T-cell immunity to viral capsid epitopes clearly contributes to the development of demyelinating disease in SJL mice. These results are consistent with the contribution of certain CD8^+^ T cell and Th17 responses to the pathogenesis of TMEV-induced demyelination [65,191].

The cellular activation status affects the levels of TMEV infection and/or replication, and the increased viral loads in turn elevate the level of inflammatory responses in a continued loop [61]. TMEV replication in infected cells is dependent on the activation of NF-κB and partially MAP kinase. In addition, treatment of macrophages with LPS, a strong inducer of innate cytokine responses, increases viral replication, and persistence in the CNS, leading to the pathogenesis of demyelination [48,59,61,218]. Several TLRs are associated with the production of inflammatory cytokines via activation of NF-κB following TMEV infection [48,67,70,73,98]. In addition, the activation of NF-κB is required for TMEV infection and replication [61]. Furthermore, the initial proinflammatory cytokines, such as TNF-α, IL-6, and IL-1, produced in virus-infected cells may further activate NF-κB in adjacent cell populations to facilitate enhanced TMEV infection and replication (Figure 2). Due to the reduction of TLR-mediated signals by blocking TLR activation or NF-κB activation during viral infections, these signals are likely associated with the pathogenesis of demyelinating disease [61,66,73,219]. In addition, TMEV infections and replications are greater in cells of susceptible mice than in cells of resistant mice, and lead to the production of excessive cytokines and TLR signals promoting the pathogenesis of demyelinating disease [67,70,73].

### 5.3. Relevance of TMEV-IDD in Understanding Other Chronic Viral Inflammatory Diseases

The significance of the TMEV system as an infectious model for MS is manifold. TMEV-induced demyelination [9,10,13,14] serves as an excellent infectious model for the following reasons: (1) wildtype isolates from infected mice, without attenuation, result in chronic demyelinating disease; (2) chronic pathological involvement is limited to the white matter of the CNS; (3) myelin breakdown is directly related to the clinical symptoms such as gait spasticity and urinary incontinence; (4) demyelination is primarily associated with cell-mediated immune responses; (5) strong autoimmunity to myelin antigens is induced following the initial demyelination by virus-specific T cells [14,30]. Susceptible SJL mice preimmunized with viral peptides or UV-inactivated TMEV prior to viral infection preferentially mount protective CD4^+^ T cell (Th1) type responses, which in turn subsequently prevent the development of pathogenic Th17 cell responses [65,127]. The development of early protective T cell and/or antibody responses reduces the viral load and the consequent pathogenesis. The ratio between the protective antiviral immune responses and the level of viral load has an inverse relationship for the development of TMEV-induced demyelinating disease [136]. Therefore, excessive production of initial inflammatory cytokines due to the high viral load, reflecting high permissiveness and viral replication in susceptible mice, appears to play a pivotal role in the pathogenesis of TMEV-induced demyelinating disease [38]. The increased cytokine production and viral load are facilitated by TLR-mediated signals, including NF-κB activation, because the reduction of these signals by blocking TLR activation or NF-κB activation during viral infections hinders the pathogenesis of demyelinating disease [61,66,73,219]. Therefore, inhibition of either TLR signals or inhibitors of NF-κB may be helpful in controlling the development of TMEV-induced demyelinating disease. It is also interesting to note that the presence of antiviral cytokines such as type I IFNs by exposing TLR ligands prior to viral infection could inhibit the development of TMEV-induced demyelinating disease [63]. In addition, the reduction of IL-6 or IL-17A in genetically modified mice or by treating mice with anticytokine antibodies prevents the development of TMEV-induced demyelination [63,65,75,83]. Therefore, functional inhibition of key inflammatory cytokines, such as IL-6, IL-1β, and/or IL-17, which are critical for the development of pathogenic T cell responses and viral persistence, may prevent the pathogenesis of TMEV-induced demyelinating disease.

The pathogenic mechanisms of TMEV-IDD discussed above may be applicable to many other chronic inflammatory diseases associated with viral infections. For example, infections with closely related viruses, such as Coxsackie viruses are known to induce various chronic organ-specific immune-mediated inflammatory diseases in animals and humans [220]. It may also be applicable to the pathogenesis of other RNA viruses, such as Corona viruses, which also induce various neurological symptoms and autoimmune-like diseases, including cytokine storms linked to excessive production of innate immune responses [221,222]. Such excessive innate immune responses by chronic viral infections may also be associated with the pathogenesis of systematic lupus erythematosus [30]. Therefore, the TMEV-IDD system with well-defined pathogenic mechanisms may provide valuable information in understanding other virus-induced inflammatory diseases.

## Figures and Tables

**Table 1:** Properties of major mouse strains and transgenic mice used in this review.

**Mouse Strains**	**Background Genes**	**MHC**	**Susceptibility**	**Th Epitopes**	**CTL Epitopes**
SJL	SJL	H-2^s^	Yes	VP2_72__−__86_, 3D_21__−__36_, VP3_24__−__37_, VP1_233__−__250_	VP3_159__−__166_, VP3_173__−__181_, VP1_11__−__20_
C57BL/6 (B6)	B6	H-2^b^	No	VP2_206__−__220_, VP4_25__−__38_,	VP2_121__−__130_
B10.S	B10	H-2^s^	No/weak	VP2_72__−__86_, 3D_21__−__36_, VP3_24__−__37_, VP1_233__−__250_	VP3_159__−__166_, VP3_173__−__181_, VP1_11__−__20_
B6.S	B6	H-2^s^	No/weak	VP2_72__−__86_, 3D_21__−__36_, VP3_24__−__37_, VP1_233__−__250_	VP3_159__−__166_, VP3_173__−__181_, VP1_11__−__20_
(SJLxB6)F1	SJL + B6	H-2^s^/H-2^b^	No/weak	VP2_206__−__220_, VP4_25__−__38_, VP2_72__−__86_, 3D_21__−__36_	VP2_121__−__130_, VP3_159__−__166_, VP3_173__−__181_, VP1_11__−__20_
**Transgene**	**Background Genes**	**MHC**	**Susceptibility**	**Th Epitopes**	**CTL Epitopes**
VP2-TCR-Tg	SJL	H-2^s^	>Yes	>>>VP2_72__−__86_,	<<VP3_159__−__166_, VP3_173__−__181_, VP1_11__−__20_
TMEV P1-Tg	SJL	H-2^s^	No	3D_21__−__86_	
TMEV P2/P3-Tg	SJL	H-2^s^	Yes	VP2_72__−__86_, VP3_24__−__37_, VP1_233__−__250_	VP3_159__−__166_, VP3_173__−__181_, VP1_11__−__20_

## Abbreviations

MS	multiple sclerosis
CNS	central nervous system
TMEV	Theiler’s murine encephalomyelitis virus
TMEV-IDD	TMEV-induced demyelinating disease
PCR	polymerase chain reaction
PFU	plaque-forming unit
PI	post-infection
S mix	peptide mix derived from structural proteins
NS mix	peptide mix derived from nonstructural proteins
Tg	transgenic mice
TCR	T cell receptor
MHC	major histocompatibility complex
